# Percutaneous Cryoablation of Clinical T1b Renal Tumors: A Tertiary Cancer Center Experience

**DOI:** 10.1007/s00270-026-04381-y

**Published:** 2026-03-17

**Authors:** Mohamed E. Abdelsalam, Ahmed Awad, Francis Kang, Zhongya Wang, David Irwin, Peiman Habibollahi, Bruno C. Odisio, Thomas Lu, Zeyad A. Metwalli, Stephen R. Lee, Jose A. Karam, Surena F. Matin, Kamran Ahrar

**Affiliations:** 1 Unit 1471, Department of Interventional Radiology, The University of Texas MD Anderson Cancer Center, 1515 Holcombe Boulevard, Houston, TX 77030 USA; 2 Department of Biostatistics, The University of Texas MD Anderson Cancer Center, Houston, TX USA; 3 Department of Urology, The University of Texas MD Anderson Cancer Center, Houston, TX USA

**Keywords:** Ablation, Outcomes, Survival rates

## Abstract

**Purpose:**

To evaluate the oncologic and survival outcomes of patients who underwent cryoablation (CA) for T1b renal masses.

**Materials and Methods:**

This retrospective observational cohort study was conducted in accordance with the STROBE guidelines. All patients with renal tumors from 4.1 cm to 7 cm in our renal ablation registry who underwent CA between January 2008, and June 2024 were included in our study. We excluded patients with renal tumors < 4 cm and patients who underwent microwave or Radiofrequency ablation, for T1b lesions. We compiled data on age, sex, tumor location and size, histopathology, procedure-related variables, and outcomes. The Kaplan–Meier methodology was used to estimate survival probabilities.

**Results:**

Fifty-one patients underwent 52 CA for 51 renal tumors. Overall, 39% of patients had a concomitant non-renal malignancy. The median tumor diameter was 4.5 cm. The median RENAL nephrometry score was 5. Most tumors (82%) were proven to be RCC by biopsy. Twenty-three patients underwent only cryoablation and 28 underwent a combination of embolization and cryoablation. Two patients (4%) experienced recurrence at the ablation site (30 months and 41.5 months after the ablation). Among the 27 patients with de novo histologically proven renal cell carcinoma included in the survival analysis, the median overall survival duration was 6.1 years, and the 5-year Local recurrence, disease and metastatic free survival rates were 87%, 83%, and 96%, respectively.

**Conclusion:**

CA (with and without embolization) is safe and effective in patients with clinical T1b RCC who are not candidates for surgery. Cryoablation offers durable local oncologic control and favorable long-term survival outcomes in appropriately selected patients.

**Graphical Abstract:**

**Figure:**
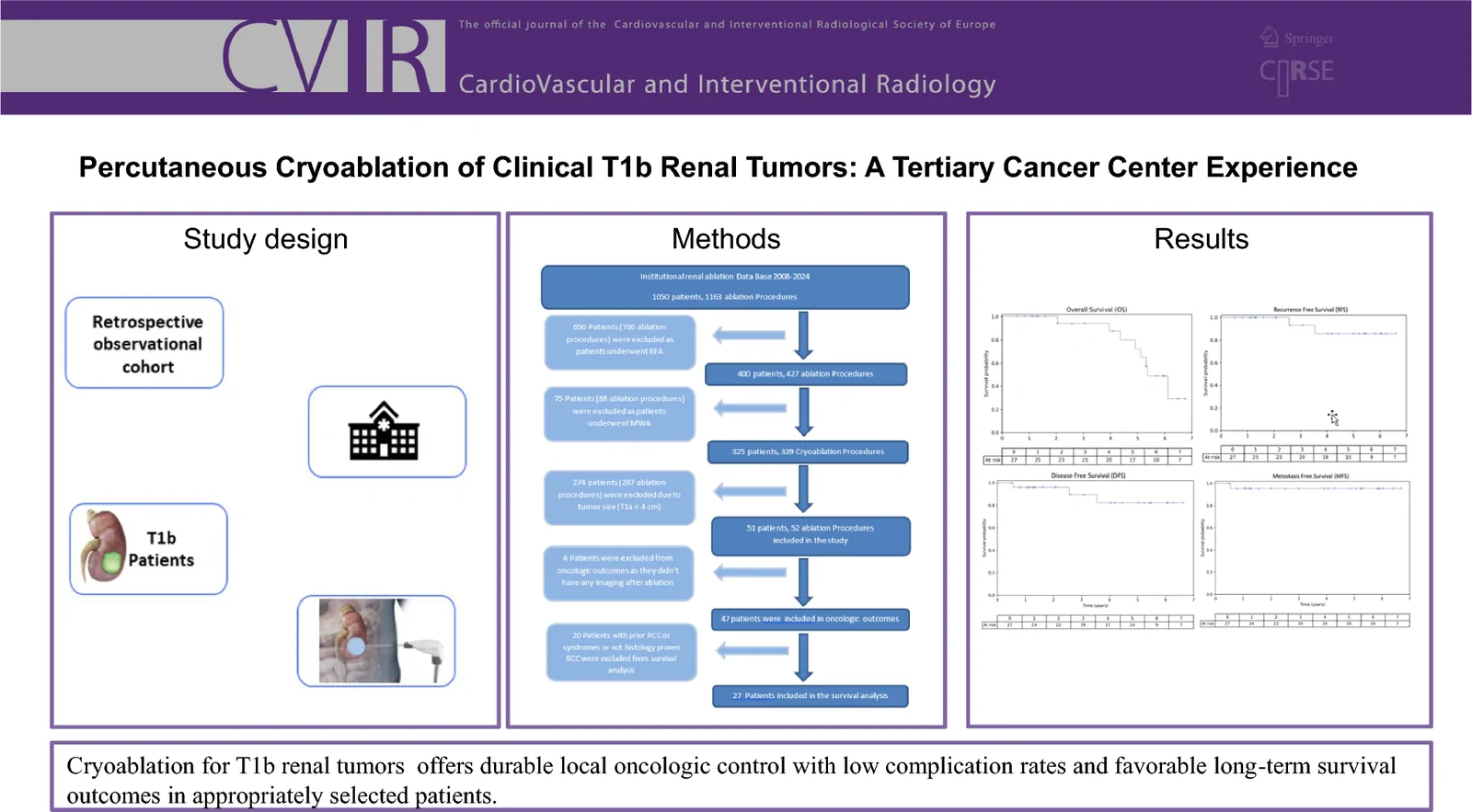

## Introduction

Minimally invasive percutaneous thermal ablation (TA) has demonstrated favorable oncologic efficacy for patients with clinical T1a renal tumors (i.e., renal tumors with a diameter ≤ 4.0 cm) [1–9]. Given this favorable efficacy, various clinical societies have acknowledged TA as a viable treatment for patients with localized T1a tumors [10–14]. Some studies have explored the feasibility of ablation techniques for larger renal masses, specifically T1b renal tumors (i.e., renal tumors with a diameter of 4.1 cm to7.0 cm) [4, 7, 15–18]. Among available ablative technologies, cryoablation has yielded encouraging oncologic outcomes for patients with T1b renal tumors [15–17], leading to interest in this approach as a potential treatment option for patients with disease deemed inoperable or those who elect not to undergo surgery.

Despite this interest, thermal ablation for T1b renal lesions has not been incorporated into the American Urological Association or the European Association of Urology clinical practice guidelines, likely due to the limitations and findings of early thermal ablation studies, which primarily investigated radiofrequency ablation, the predominant modality at the time. Preliminary evidence suggests that percutaneous cryoablation can overcome the limitations of radiofrequency ablation. Cryoablation offers large tumor coverage within the ablation zone, leading to favorable local oncologic control with low rates of residual disease and recurrence [15–18]. While complications following CA of larger tumors remain a concern [19, 20], early findings suggest that the ablation of T1b tumors can be safe with appropriate periprocedural care [16, 17]. Despite these encouraging results, there’s limited evidence and more research is needed to fully establish the role of percutaneous cryoablation in treating T1b renal cell carcinoma (RCC). This retrospective study aimed to comprehensively evaluate the long term -oncologic outcomes and survival rates of patients who underwent cryoablation for T1b renal masses.

## Patients and Methods

### Patient Selection

This retrospective observational cohort study was conducted in accordance with the STROBE (Strengthening the Reporting of Observational Studies in Epidemiology) guidelines. We retrospectively reviewed the medical records of patients included in our renal ablation registry who underwent cryoablation from January 2008 (when our institution began performing cryoablations) to June 2024. The study was approved by the institutional review board, and a waiver for informed consent was granted. The study included patients with T1b renal masses (i.e., renal tumors with a diameter of 4.1 cm to 7 cm) who underwent percutaneous image-guided cryoablation. Patients with T1a renal tumors (i.e., renal tumors with a diameter < 4 cm) and patients who underwent other ablation modalities, such as microwave ablation or radiofrequency ablation, for T1b lesions were excluded.

Patients were referred to the interventional radiology outpatient clinic following a multidisciplinary discussion, typically led by the attending urologist. Ablation was considered a preferred treatment modality in patients with significant comorbidities or poor kidney functions, those with another non-renal primary malignancy, patients who declined active surveillance, or those who elected ablation as an alternative to surgical management. Prior to treatment, an interventional radiologist evaluated patients for candidacy for ablation with a thorough assessment of their medical history, radiologic studies, and laboratory findings.

### Technique

#### Cryoablation Technique

Our ablation technique was described previously [21, 22]. Briefly, in our practice, we use cryoablation for renal lesions with a maximum diameter > 3.5 cm. The procedure is performed with the patient under general anesthesia using imaging guidance with either  computed tomography (CT) or magnetic resonance imaging (MRI). Patients whose lesions have a diameter > 5 cm or exhibit marked hypervascularity typically undergo transarterial embolization before ablation to reduce the risk of bleeding [23, 24] and possibly optimize cryoablation success through better delineation of the tumor margin. The number, size, and spatial arrangement of the cryoprobes are selected to ensure complete tumor coverage, with the ablation zone extending approximately 0.5 cm to 1.0 cm beyond the tumor boundary to optimize treatment margins. Patients without a histologic diagnosis of the tumor undergo an image-guided percutaneous biopsy in the same session immediately before the ablation and after embolization in combined approach cases.

When anatomically adjacent critical structures are at risk, adjunctive protective techniques such as hydrodissection or pyeloperfusion are performed. For tumors not clearly visualized with CT, MRI-guided cryoablation is conducted using a 1.5-Tesla scanner with balanced steady-state free precession sequences to enable real-time visualization and planning [22]. These procedures use MRI-compatible cryoablation  systems. Following treatment, patients immediately undergo post-ablation contrast-enhanced imaging, which is used to assess technical success, complete ablation of the lesion, and potential acute complications. Radiologic surveillance is performed at standardized intervals during the first 24 months after the procedure, followed by annual imaging assessments to monitor for recurrence. Our follow-up clinical practice has evolved over time in response to our research and the accumulation of published scientific evidence. Earlier in the study period, follow-up imaging was routinely performed at 1, 3, and 6 months, followed by 1 year and annually thereafter. In more recent practice, follow-up imaging has been streamlined to assessments at 6 months and then annually.

#### Embolization Technique

At our institution, patients whose tumors are > 5 cm or exhibit marked hypervascularity typically undergo transarterial embolization prior to ablation. The primary objective of embolization is to mitigate the risk of hemorrhagic complications during cryoablation rather than to confer therapeutic oncologic benefit [23, 24]. The pre-ablation embolization is performed one day before or on the day of the ablation procedure. A 5F catheter is advanced through the femoral or radial artery to the renal artery of the involved kidney, and a baseline digital subtraction angiogram is acquired. Then, intraprocedural CT angiography of the main renal artery is performed to better delineate the tumor and its arterial supply. Subsequently, selective catheterization of the arterial branches feeding the tumor is performed using a microcatheter advanced through the 5F catheter. Embolization is conducted using alcohol or particles until flow stasis is achieved. After embolization, a final digital subtraction angiogram of the main renal artery is acquired to confirm the complete and successful devascularization of the tumor and preservation of perfusion to uninvolved renal parenchyma.

### Data Collection

From each patients’ medical records, we gathered the following data: age, sex, lesion size, lesion laterality, RENAL nephrometery score, histopathologic subtype, nuclear (Fuhrman) grade, and prior or concurrent primary malignancies. Procedural variables included the ablation modality, guidance modality (CT or MRI), employment of adjunctive protective techniques, and ablation success. Procedural complications were categorized using Clavien-Dindo classification. Post-treatment radiologic images were assessed for incomplete ablation or residual tumor within the ablation zone, new renal lesions outside the treated area, and distant metastases originating from RCC. Patient vital status, and if, applicable, cause and date of death were documented. To minimize selection and referral bias, all consecutive eligible patients from a prospectively maintained renal ablation registry were included. Missing histopathologic data, including absent biopsy or Fuhrman grade in a minority of cases, were retained to reflect real-world practice. Variable follow-up duration was addressed through time-to-event analyses with appropriate censoring.

### Definitions of Outcomes

We defined technical success as successful probe placement and complete execution of the planned ablation. Residual tumor was defined as contrast uptake within the ablation zone on the initial follow-up cross-sectional imaging. Local tumor recurrence was defined as contrast uptake within the ablation zone that was not depicted on prior imaging or as histopathologic confirmation of viable tumor cells within the treated zone in a post-ablation biopsy sample. Cases of residual or recurrent disease were evaluated for repeat ablation, active surveillance, or surgery.

Definitions of survival outcomes were extracted from the American Urological Association guidelines for managing small renal masses [25]. Overall survival (OS) was defined as the proportion of living patients at the time of data collection; local recurrence-free survival (LRFS), as the proportion of patients without residual or recurrent disease within the ablation zone; metastasis-free survival (MFS) as the proportion of patients without metastatic disease; and disease-free survival (DFS) as the proportion of patients without evidence of local or distant disease. Cancer-specific survival (CSS) was defined as the proportion of patients who did not die of their renal cancer.

### Data Analysis and Statistics

Descriptive statistics were used to summarize demographic, clinical, tumor, procedural, and histopathologic variables. Kaplan–Meier survival analysis was used to estimate OS, LRFS, DFS, MFS, and CSS. Time-to-event data were measured from the date of cryoablation to the occurrence of the relevant event. Patients with recurrent or multiple tumors, those without biopsy-proven RCC, those with genetic syndromes, and those with metastatic disease were excluded from the survival analysis. Only patients with a solitary de novo histologically confirmed RCC lesion were included in this analysis. Key potential confounders (age, RENAL score, and presence of non-renal malignancy) were collected a priori but not included in multivariable modeling given the descriptive study objective and limited event counts. Missing data (including ~ 10% without biopsy results or Fuhrman grade) were handled using an available-case approach without imputation. Kaplan–Meier analysis was selected for consistency with prior renal ablation literature; competing-risk methods were considered but not applied. Patients were censored at last known follow-up, and proportional hazards assumptions were not formally tested as no regression modeling was performed.

## Results

### Patient Characteristics

**Fig. 1:**
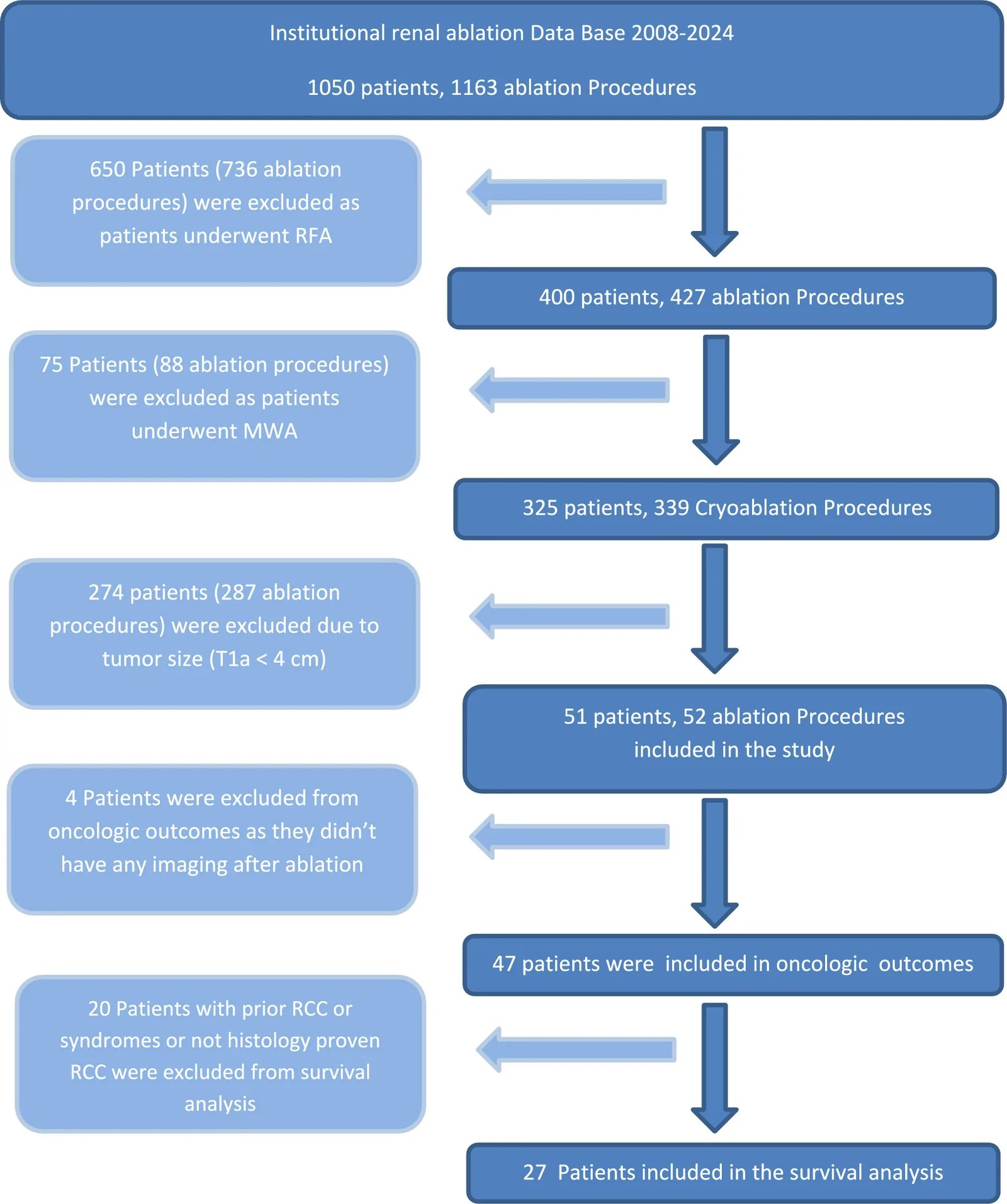
Flow Chart showing patients meeting the exclusion criteria for the study

In total, 51 patients met the predefined eligibility criteria for inclusion in this study. Figure 1 shows a flow Chart of patients meeting the inclusion and exclusion criteria for the study. The cohort included 29 men (57%) and 22 women (43%), with a median age of 72 years (interquartile range: 47.5 years–90.6 years). Notably, 20 patients (39%) had a documented history of an additional non-renal primary malignancy. Among these 20 patients, 13 had disease in remission, 6 were receiving active oncologic treatment, and one was scheduled to undergo renal ablation before the initiation of systemic therapy for the concurrent malignancy. A comprehensive summary of patient demographics, tumor characteristics and indications for ablation is summarized in Table 1

**Table 1:** Patient and tumor characteristics (N = 51)

Characteristic	n (%)
Age, years, median (range)	72 (47.5–90.6)
*Sex*	
Female	22 (43)
Male	29 (57)
*Non-renal malignancy*	
Yes	20 (39)
No	31 (61)
*Tumor size, cm, median (range)*	4.5 (4.1–6.8)
*Affected kidney*	
Left	23 (45)
Right	28 (55)
*Tumor location*	
Exophytic	14 (27)
Intraparenchymal	12 (24)
Mixed	25 (49)
*RENAL nephrometry score*	
Low (4–6)	28 (55)
Moderate (7–9)	9 (18)
High (10–12)	14 (27)
*Histopathology*	
Clear cell	33 (65)
Papillary	5 (10)
Chromophobe	4 (8)
Others	4 (8)
Not available	5 (10)
*Fuhrman grade*	
1	4 (8)
2	21 (41)
3	5 (10)
4	1 (2)
Not available	20 (39)
*Indications for Ablation (some patients have more than one indication):*	
Medical Comorbidities	30
Another non renal malignancy	20
Impaired renal functions	11
Solitary kidney	3
Patient preference for ablation	3
Prior ipsilateral partial nephrectomy	2
Genetic syndrome/bilateral renal masses	2

### Procedural Outcomes

In total, 52 cryoablation procedures were performed for 51 renal tumors among 51 patients. The median renal tumor diameter was 4.5 cm (range: 4.1 cm–6.8 cm; mean ± standard deviation: 4.7 cm ± 0.66 cm), and 28 tumors (55%) and 23 tumors (45%) were in the right and left kidneys, respectively. Fourteen tumors (27%) were classified as exophytic, 12 (24%) as intraparenchymal, and 25 (49%) as having mixed characteristics. The median RENAL nephrometry score was 5. All procedures (100%) were conducted under CT guidance.

Preprocedural transarterial embolization was performed in 28 patients. Adjunctive techniques included hydrodissection in 23 procedures (45%) and pyeloperfusion through a retrograde ureteral catheter in 4 cases (8%). Technical success was achieved in all cases.

Seven patients had Clavien-Dindo grade III or higher complications following ablation. Among these patients, 5 experienced hemorrhagic complications: 2 patients had hematomas (one perirenal and one subcapsular), both of which required angiography and embolization immediately after the ablation procedure; one patient developed obstructive uropathy due to clot formation, which required percutaneous nephroureteral stent placement; and two patients developed hematuria that required bladder irrigation. In addition, one patient experienced a post-procedural infection, which was managed with percutaneous drainage, and another patient developed acute respiratory compromise during extubation, requiring admission to the intensive care unit. Complications are classified and reported according to the 2026 modified CIRSE classification system in.Table 2 [26].

**Table 2:** Complications classified according to Modified CIRSE classification system

Pt	Complication	Management	Modified CIRSE classification
1	Perirenal Hematoma	Angiography and embolization	3a
2	Subcapsular Hematoma	Angiography and embolization	3a
3	Obstructive Uropathy secondary to blood clot	Percutaneous nephro-ureteral stent placement	3a
4	Hematuria	Bladder irrigation	3a
5	Hematuria	Bladder irrigation	3a
6	Infection	Percutaneous drain placement	3b
7	Acute respiratory compromise	ICU admission	3a

Complications were identified across both groups (with and without embolization), in lesions with low and high RENAL nephrometry scores, and in both central and exophytic tumor locations.

### Histopathologic Outcomes

Histopathologic data were available for 46 tumors. The predominant histologic subtype was clear cell RCC (65%), followed by papillary RCC (10%). Fuhrman nuclear grading was available for 31 tumors, with most of these tumors classified as grade 2(n = 21,41%). Detailed histopathologic results are provided in Table 1.

### Oncologic Outcomes

Four patients were excluded from the oncologic outcome analysis due to the absence of any post-ablation imaging. The remaining patients were monitored longitudinally through coordinated clinical and radiological follow-up assessments by both Urology and Interventional Radiology services. The median duration of follow-up was 3.0 years (range: 0.1 years–13.3 years).

Of the 47 patients included in the oncologic outcome analysis, one (2%) with a known diagnosis of Von Hippel–Lindau syndrome had initial post-procedural imaging showing persistent contrast enhancement within the ablation zone, consistent with residual disease, and subsequently underwent repeat cryoablation. Two additional patients (4%) developed local recurrence within the treated area, one at 30 months and the other at 41.5 months after the ablation procedure. Both recurrences were confirmed as clear cell RCC. There was no significant difference in local recurrence between the two groups (p = 1.00), with one recurrence observed in the cryoablation-only group and one in the combined embolization and ablation group. One of these patients underwent repeat cryoablation, and the other elected to receive care at a different facility.

### Survival Outcomes

Patients with a history of RCC, hereditary syndromes, or those without histologically confirmed RCC were excluded from the survival analysis to reduce potential confounding factors in the outcome analysis. Twenty-seven patients had a solitary de novo histologically confirmed RCC lesion and were included in the survival analysis. Survival outcomes for all patients and for patients stratified by receipt of embolization are given in Table 3. For all patients, the median OS duration was 6.1 years, and the 5-year OS rate was 75%.

**Table 3:** Survival outcomes

Survival type	Median survival duration (95% CI), years	5-year survival rate, %	*p*-value
*OS*			
All	**6.1** (4.88 – NE)	**75**	
Cryoablation	5.34 (2.02 – NE)	64	**0.69**
Cryoablation and embolization	6.10 (4.88 – NE)	81	
*LRFS*			
All	**5.28** (3.93 – NE)	**87**	
Cryoablation	4.32 (2.02 – NE)	80	**0.42**
Cryoablation and embolization	6.10 (3.93 – 6.10)	91	
*DFS*			
All	**5.28** (3.93 – 6.10)	**83**	
Cryoablation	4.32 (2.02 – NE)	80	**1.00**
Cryoablation and embolization	6.10 (3.93 – 6.10)	91	
*MFS*			
All	**5.34** (4.32 – NE)	**96**	
Cryoablation	4.32 (2.02 – NE)	100	**0.35**
Cryoablation and embolization	6.10 (3.93 – NE)	93	

CI, confidence interval; CA, cryoablation; OS, overall survival; NE, not estimable; LRFS, local recurrence–free survival; DFS, disease-free survival; MFS, metastasis-free survival

The median LRFS, DFS, and MFS durations were 5.3, 5.3, and 5.4 years, respectively. The 5-year LRFS, DFS, and MFS rates were 87%, 83%, and 96%, respectively. The CSS rate was 100%, as none of the patients died from RCC at the time of data collection.

**Fig. 2:**
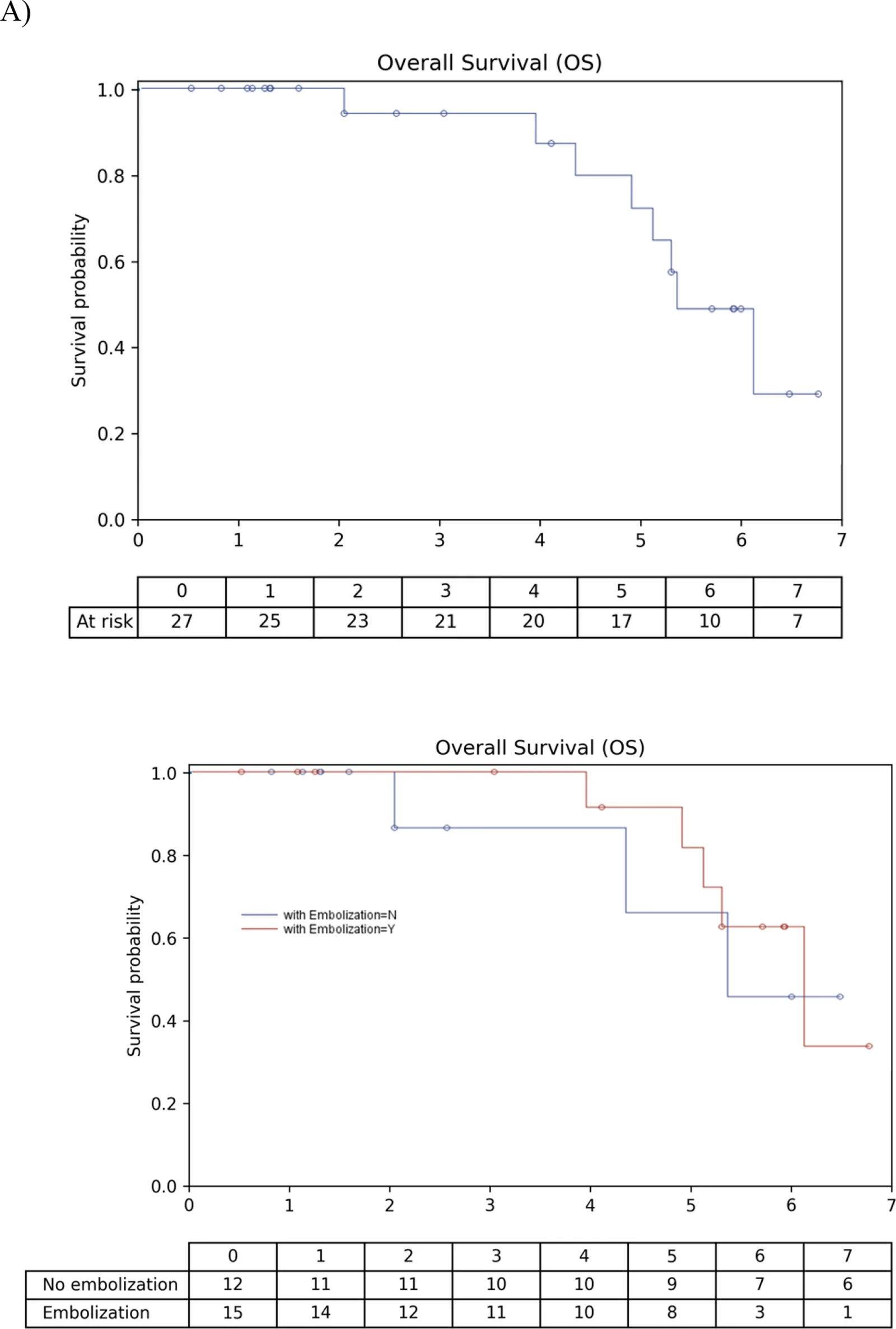

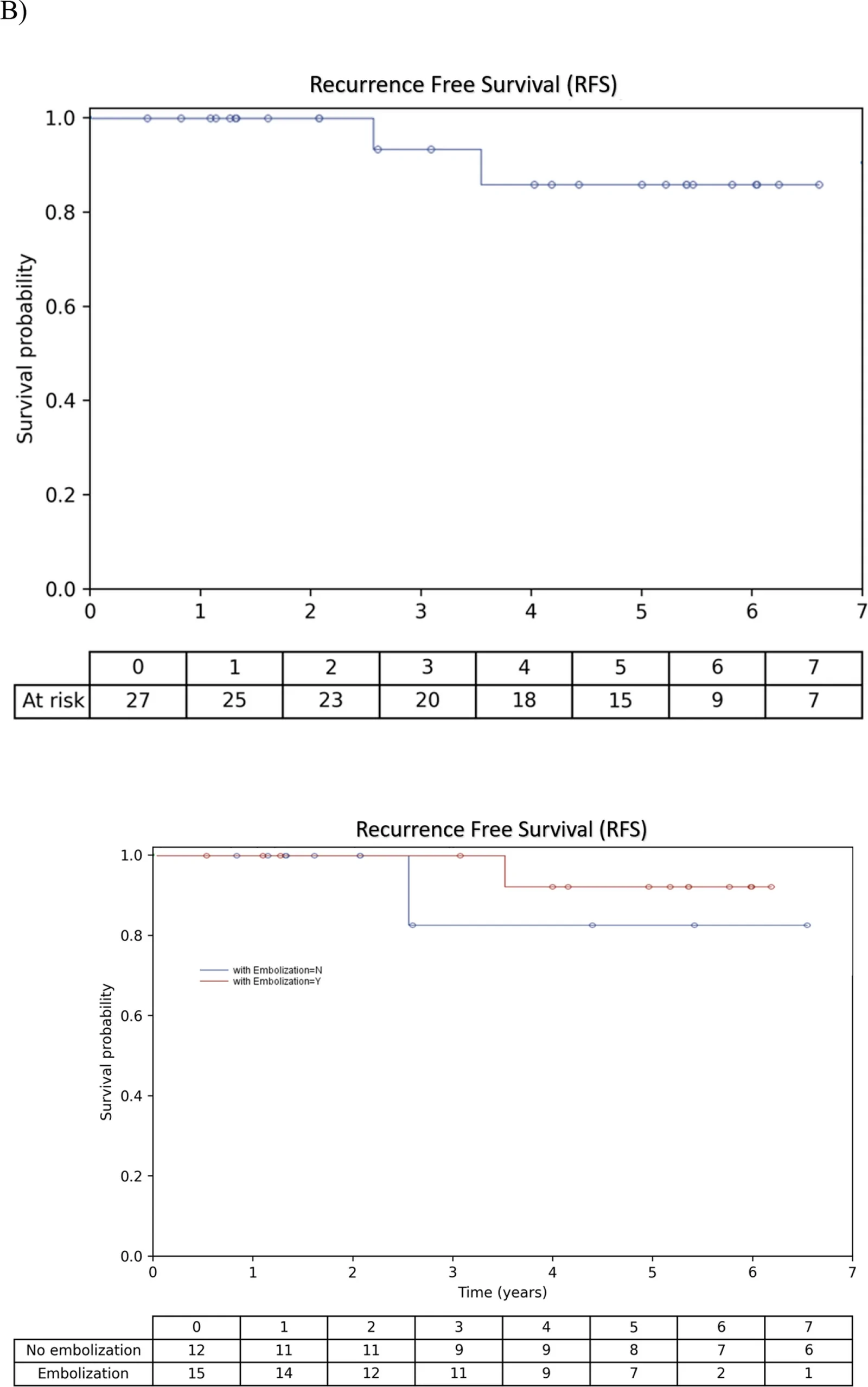

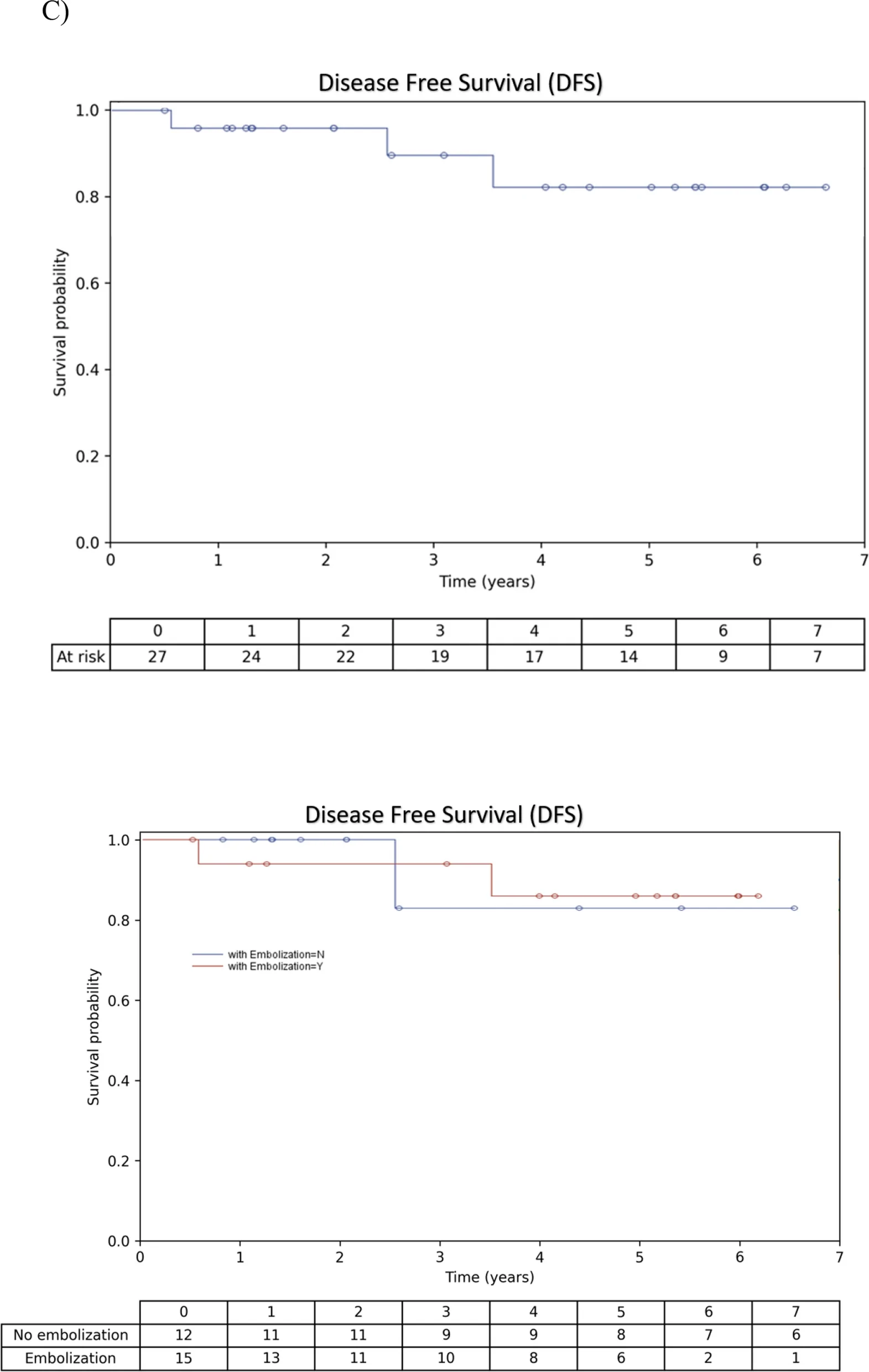

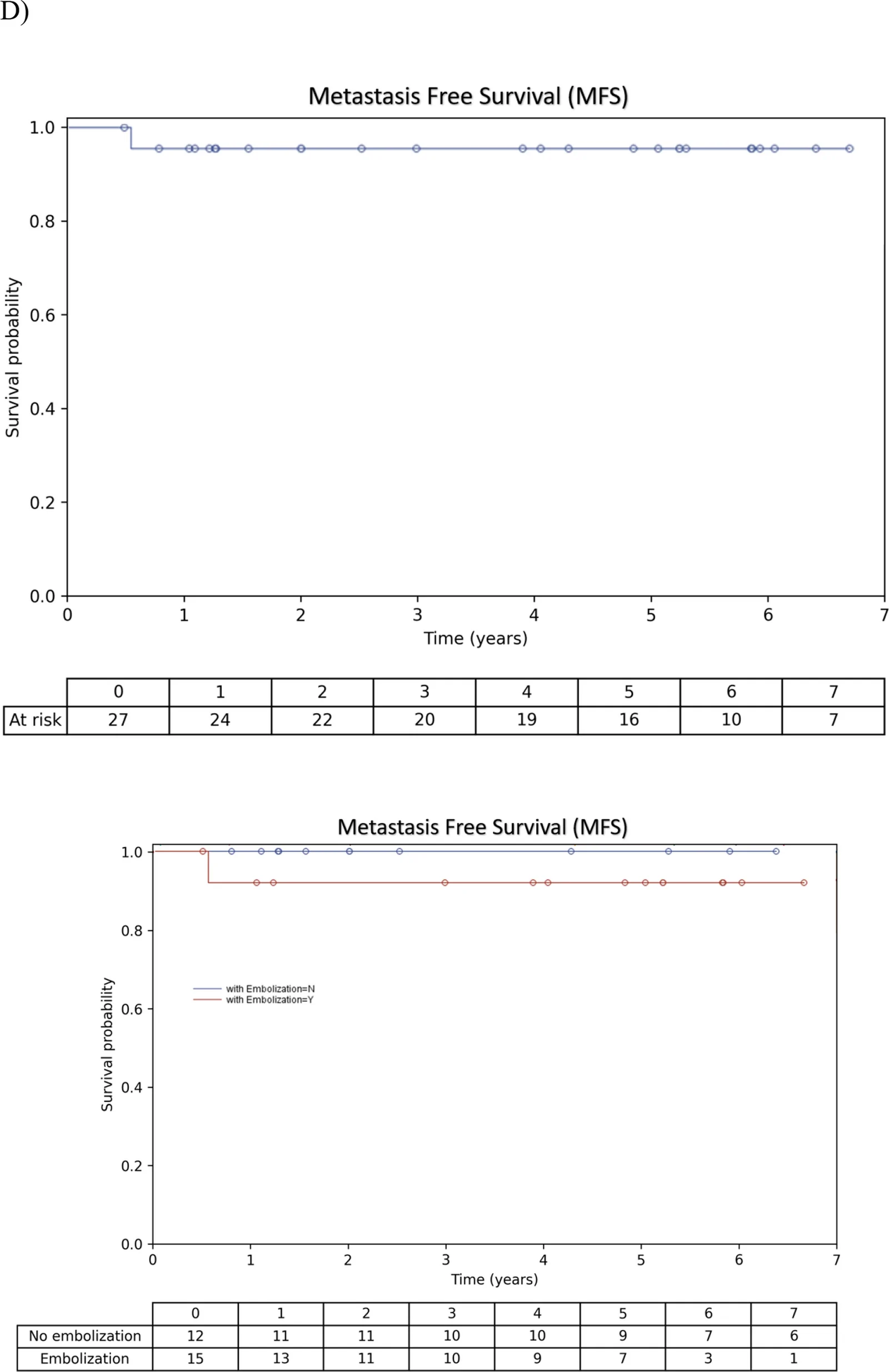
Kaplan–Meier survival curves for **A** overall survival, **B** local recurrence–free survival, **C** disease-free survival, and **D** metastasis-free survival for all patients (top) and for patients stratified by embolization status (bottom)

OS, LRFS, DFS, and MFS did not differ significantly between the patients who underwent only cryoablation and those who underwent combined embolization and cryoablation (p = 0.69, p = 0.42, p = 1.00, and p = 0.35, respectively).The Kaplan–Meier estimates of OS, LRFS, MFS, and DFS for all patients and both groups for patients stratified by receipt of embolization are shown in Fig. 2.

## Discussion

This study demonstrates a 5-year OS rate of 75% among our cohort of patients who underwent cryoablation for their T1b renal tumors. In an earlier study [27], we found that patients with RCC and concomitant primary non-renal malignancies had significant shorter OS than patients with only RCC. This explains the relatively low OS rate in the current study, as 39% of the patients had an additional non-renal malignancy. Nonetheless, the median OS duration of our entire cohort was 6.1 years.

Our  outcomes  are largely consistent with those of previous studies of cryoablation for T1b renal tumors. Our data demonstrated high oncologic efficacy, with primary efficacy rates of 98% at 6 months and 95% at 5 years. These findings are consistent with those reported by Michailidiset al. [28], who observed a primary efficacy of 95% and a secondary efficacy of 98.8% at 6 months. Similarly, King et al. reported a primary technical efficacy of 94.1% at 3 months among patients with T1b tumors in the EuRECA registry [29]. In contrast, Junker et al. [30] reported substantially lower efficacy rates, with primary and secondary efficacies of 68% and 77%, respectively. This discrepancy may be attributable to several factors, including differences in patient selection, procedural techniques, operator experience, and institutional practice patterns.

Atwell et al. [16] investigated cryoablation for 46 patients with biopsy-proven RCC, whose mean tumor diameter was 4.8 cm (range: 4.1 cm–6.4 cm). Their rate of technical success, defined as the absence of post-procedural contrast enhancement or lesion enlargement within 3 months following ablation, was 97.2%. Additionally, they reported 5-year local progression-free survival and CSS rates of 96.4% and 94%, respectively. These rates align with our study’s LRFS, MFS, and CSS rates of 87%, 96%, and 100%, respectively. Similarly, Gunn et al. [17] evaluated the outcomes of 37 patients who underwent cryoablation for T1b lesions; these patients had a mean tumor size of 4.7 cm and a median follow-up time of 16.4 months (range: 0.03 months–102.6 months). Using criteria consistent with those used by Atwell et al. [16], the authors reported a technical success rate of 88.2% and 3-year OS, RFS, and CSS rates of 77.6%, 62.6%, and 100%, respectively. These results are also concordant with the survival outcomes we found in our current study.

The 5-year survival rates of patients who underwent cryoablation for T1b renal tumors in the present study were consistent with those previously reported for patients who underwent partial nephrectomy for such tumors [31–34]. For example, in a study of 119 patients who underwent partial nephrectomy or percutaneous cryoablation for T1b renal tumors, Aikawa et al. [31] found that the cryoablation group had a higher rate of local recurrence but found no significant differences in OS (*p* = 0.11), MFS (*p* = 0.23), or CSS (*p* = 0.77) between the 2 groups. The partial nephrectomy group’s 5-year OS, LRFS, MFS, and CSS rates were 96%, 95%, 95%, and 99%, respectively, closely matching the rates observed in our study.

This study is subject to the limitations inherent to a retrospective design, including potential selection bias, as patient inclusion was dependent on clinical decision-making and referral patterns rather than randomization. Additional sources of bias may include information bias related to documentation accuracy and imaging follow-up, as well as unmeasured confounding variables that could not be fully accounted for despite standardized data collection. In addition, our study reflects the experience of a single institution with a relatively small patient cohort; our results can’t be generalized given the single-institution design, with potential influence from institution-specific standards of practice e.g. embolization techniques. A multi-institutional study with a larger sample size would provide a more comprehensive view of the broader patient population and have more robust statistical power. Also, not all cases in the present study were histologically confirmed to be RCC. Furthermore, as a single-arm study without a control group, it could not directly compare outcomes achieved with ablation and those achieved with alternative treatments.

## Conclusion

When combined with adjunctive procedures, cryoablation (with and without embolization) is a safe and effective treatment for patients with T1b renal tumors who are not candidates for surgery. Cryoablation offers durable local oncologic control with low complication rates and favorable long-term survival outcomes in appropriately selected patients. These findings should be interpreted in the context of the inherent limitations of the study.
